# Molecular Detection of Multidrug Resistant *Salmonella* Species Isolated from Broiler Farm in Bangladesh

**DOI:** 10.3390/pathogens9030201

**Published:** 2020-03-09

**Authors:** Shanzida Binte Alam, Muket Mahmud, Rafiya Akter, Mahadi Hasan, Abdus Sobur, KHM Nazmul Hussain Nazir, Ayman Noreddin, Tanvir Rahman, Mohamed E. El Zowalaty, Marzia Rahman

**Affiliations:** 1 Department of Microbiology and Hygiene, Bangladesh Agricultural University, Mymensingh 2202, Bangladesh; shanzidashanu20@gmail.com (S.B.A.); muketvet@gmail.com (M.M.); rafia.karim06@gmail.com (R.A.); hasan.48603@bau.edu.bd (M.H.); soburvetbau@gmail.com (A.S.); nazir@bau.edu.bd (K.N.H.N.); tanvirahman@bau.edu.bd (T.R.); 2 University of California, Irvine, CA 92697, USA; anoreddin@sharjah.ac.ae; 3 Infectious Diseases and Anti-Infective Therapy Research Group, Sharjah Medical Research Institute and College of Pharmacy, University of Sharjah, Sharjah 27272, UAE; 4 Zoonosis Science Center, Department of Medical Biochemistry and Microbiology, Uppsala University, Uppsala SE-75185, Sweden

**Keywords:** multidrug resistant, *Salmonella*, poultry, litter, feed, integron, one health, Bangladesh

## Abstract

Multidrug resistant (MDR) *Salmonella* are a leading cause of foodborne diseases and serious human health concerns worldwide. In this study we detected MDR *Salmonella* in broiler chicken along with the resistance genes and class 1 integron gene *intl1*. A total of 100 samples were collected from broiler farms comprising 50 cloacal swabs, 35 litter and 15 feed samples. Overall prevalence of *Salmonella* was 35% with the highest detected in cloacal swabs. Among the *Salmonella*, 30 isolates were confirmed as *S. enterica* serovar Typhimurium using molecular methods of PCR. Disk diffusion susceptibility test revealed that all the *Salmonella* were classified as MDR with the highest resistance to tetracycline (97.14%), chloramphenicol (94.28%), ampicillin (82.85%) and streptomycin (77.14%). The most prevalent resistance genotypes were *tetA* (97.14%), *floR* (94.28%), *bla_TEM-1_* (82.85%) and *aadA1* (77.14%). In addition, among the MDR *Salmonella,* 20% were positive for class 1 integron gene (*intl1)*. As far as we know, this is the first study describing the molecular basis of antibiotic resistance in MDR *Salmonella* from broiler farms in Bangladesh. In addition to *tetA, floR, bla_TEM-1_*, *aadA1* and *intl1* were also detected in the isolated MDR *Salmonella.* The detection of MDR *Salmonella* in broiler chicken carrying *intl1* is of serious public health concern because of their zoonotic nature and possibilities to enter into the food chain.

## 1. Introduction

Poultry farming is a profitable agricultural business globally, in Bangladesh; livestock and poultry contribute approximately 1.47% of the total GDP of the country [1]. In the last decade, tremendous development has been achieved in this sector in Bangladesh [2]. Poultry products including eggs and meat are excellent sources for humans’ daily protein requirement. The advancement of poultry production is being seriously hampered by management factors, such as biosecurity measures and infectious diseases [3]. Among the infectious diseases, salmonellosis caused by MDR *Salmonella* is a major constrain for poultry industries in Bangladesh [4] and is a worldwide epidemic causing millions of illness and deaths annually [5].

The chicken-adapted *Salmonella* serovar Gallinarum biovar *Pullorum* and Gallinarum are responsible for avian salmonellosis, e.g., pullorum disease and fowl typhoid, respectively [6]. In poultry, Salmonellosis is often associated with high mortality up to 90%, thus causing severe economic losses [7]. *S. enterica* serovar Typhimurium normally colonizes the cecum and is commonly found asymptomatic in cloaca in older chicks [7]. Day old chicks infected with *Salmonella* may show intestinal pathology and inflammatory responses [8]. Although birds have no obvious disease symptoms, *S. enterica* serovar Typhimurium can cause systemic metabolic changes in birds affecting production [9]. Human salmonellosis outbreaks are mostly associated with the consumption of poultry products contaminated with *S. enterica* serovar Typhimurium and *S. enteritidis* [10]. Therefore, the occurrence of *Salmonella,* particularly *S. enterica* serovar Typhimurium, in broiler farms is a public health concern as they can transmit to the food chain. 

Modern food animal production, specifically broiler chicken production, is directly dependent on the use of antibiotics as growth promoters [11]. Antibiotics are also used in broiler chicken for treatment purposes. The escalated use of antibiotics causes the development of resistance through selection pressure [12]. Antimicrobial resistance (AMR) is an increasingly serious public health challenge [13]. It is speculated that if measures are not taken properly, by 2050 AMR will cause 300 million human deaths, financial losses will be equivalent to 100 trillion USD and an 11% fall in animal production [14]. By far, most of the affected countries will be the low and middle-income countries (LMICs) in Africa and Asia [15]. Bangladesh is located in the World Health Organization’s Southeast Asia region which is assessed as having a high risk of AMR [16].

Bacteria can acquire resistance genes through mobile genetic elements such as plasmids, transposons, integron, and insertion sequences (IS) elements [17]. Due to their mobile nature, these genetic elements can easily spread horizontally across many species of bacterial populations [18]. Humans can get exposed to antibiotic resistant bacteria through consumption of contaminated meat and eggs, or through direct transmission from colonized animals or manure and litter [19]. Because of their zoonotic nature, there are possibilities for the transmission of MDR *Salmonella* from poultry to humans throughout the food chain [20,21,22]. In recent years, the development of MDR among foodborne pathogens, such as *Salmonella* spp., have been associated with an increase in human mortality, and longtime hospitalization due to therapy failure [22].

The isolation of *Salmonella* from poultry was previously reported in Bangladesh, however, none of these studies focused on the molecular detection of resistance genes [23,24,25,26]. However, resistance genes were detected in *Escherichia coli* isolated from cloacal swabs of boiler chicks in Bangladesh [27,28]. Moreover, a study in Bangladesh, detected resistance genes in *E. coli* and *Salmonella* from dairy farms [29]. Another study detected integrons in *Salmonella* from chicken feces [30]. Therefore, we investigated the prevalence of resistance genes including integrons responsible for multidrug resistance in *S. enterica* serovar Typhimurium in broiler farms using different sample types (cloacal swabs, litter and feed).

## 2. Results

**Figure 1:**
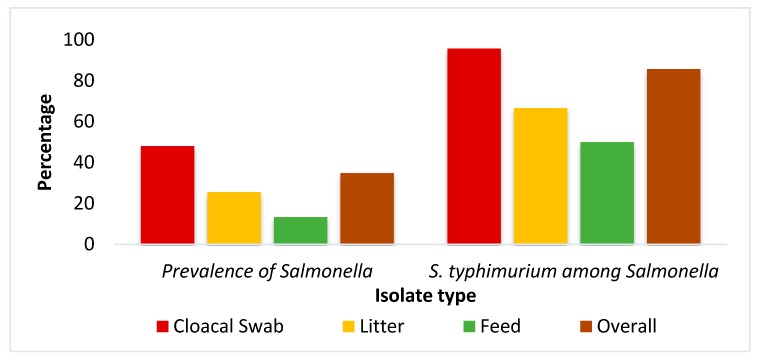
Prevalence of *Salmonella* spp. and *S. enterica* serovar Typhimurium in broiler farms.

Among the 100 samples analyzed, 35 (35%) were found to be positive for the presence of *Salmonella* as evidenced from the isolation on selective media, followed by Gram staining, biochemical tests, and *invA* gene-targeted PCR (Figure 1). The prevalence of *Salmonella* was significantly (Chi-square test, *p* = 0.017) higher in cloacal samples (48%, n = 24) compared to all types of samples. The confirmed 35 isolates were inoculated into motility indole urea medium and 30 samples were found to be motile, suggesting that these motile *Salmonella* would be *S. enterica* serovar Typhimurium. Motile and non-motile *Salmonella* were also differentiated by PCR amplification of *fliC* gene specific for motile *S. enterica* serovar Typhimurium. All 30 motile isolates were also found to be positive for the *fliC* gene, confirming them as *S. enterica* serovar Typhimurium. The detection of *S. enterica* serovar Typhimurium was found to be the highest significantly in cloacal sample type (95.8%, n = 23) as compared to litter and feed samples (Chi-square test, *p* = 0.034). 

From the antibiogram study, it was found that the isolated *Salmonella* were highly resistant to tetracycline (97.1%) followed by chloramphenicol (94.3%), ampicillin (82.9%), and streptomycin (77.1%), while the highest susceptibility rate was observed against cefixime (100%), followed by ertapenem (94.3%) and ciprofloxacin (85.7%) (Table 1). *Salmonella* from cloaca always showed a peak of resistance against all antibiotics and a statistically significant relationship was found for tetracycline, chloramphenicol and ampicillin. All 35 identified *Salmonella* were MDR with the highest 80% (n = 28) against three antibiotics classes, and only two isolates showed MDR phenotype against five antibiotic classes (Table 2). 

It was found that 34 (97.1%) out of 35 isolates were positive for tetracycline resistance gene *tetA*. The prevalence of *floR*, *bla_TEM-1_*, and *aadA1* in *Salmonella* were 94.3%, 82.9%, and 77.1%, respectively, from different sources (Table 3). *Salmonella* isolated from cloacal swabs showed a statistically significant peak prevalence for all the resistance genes similar to their phenotype. Among the 35 isolates, 7 (20%) were found positive for class 1 integron gene *intl1*. Although cloacal isolates showed the highest prevalence of *intl1*, no statistically significant relationship was found when compared with isolates originated from litter and feed. These seven isolates carried multiple resistance genes (Table 4).

## 3. Discussion

Avian salmonellosis is a major threat for poultry industries since it is capable of causing heavy economic losses through mortality and reduced production. *Salmonella* are also important foodborne zoonotic pathogens. In this study, among the samples analyzed, 35% (35/100) were found to be positive for the presence of *Salmonella* as confirmed by PCR. Earlier, Al Mamun et al. [26] reported the prevalence of Salmonellosis in poultry as 23.53%, lower than the present findings, and Mahmud et al. [23] reported a prevalence of 37.9% in Bangladesh, which is quite similar to the current findings. The prevalence of *Salmonella* was significantly the highest in cloacal swabs (48%). Previously a study in Bangladesh, showed the highest rate of *Salmonella* occurrence in cloacal swabs (32%) among different samples of poultry [31] and another study found 48% *Salmonella*, similar to our result [25]. Litter (25.7%) was also found to be contaminated with *Salmonella* in the present study, whereas Islam et al. [32] reported 66.6% *Salmonella* in litter from broiler farms. *Salmonella* could be either motile or non-motile. The motile *Salmonella* are mostly associated with food products and are the major causes of salmonellosis in humans worldwide [33]. In the current study, 30 out of 35 *Salmonella* isolates were confirmed as motile *Salmonella*, i.e., *S. enterica* serovar Typhimurium by growing on motility indole urea agar and by *fliC gene* detection through PCR. Barua et al. [24] reported the presence of motile *Salmonella* in commercial broiler chicken farms as 11%. Another study in Bangladesh revealed 15.91% *S. enterica* serovar Typhimurium among the identified *Salmonella* [25]. Detection of *S. enterica* serovar Typhimurium in poultry implies the possibility of *S. enterica* serovar Typhimurium transmission via poultry originated food and may lead to foodborne illness in human [22].

These observed variations in the prevalence of *Salmonella* may be linked with various management factors such as biosecurity, hygiene, and sanitation of the farms. The presence of *Salmonella* spp. in cloacal swabs of healthy broiler chicken provides the evidence of persistent intestinal colonization of *Salmonella* spp. of the individual bird [34]. Moreover, their presence in cloacal swabs and poultry litter indicates that poultry droppings may act as vehicles for shedding *Salmonella* spp. to other birds [34]. In addition, poultry litter aeration can be a vital risk factor for spreading pathogens like *Salmonella* and can contaminate farm environments, causing birds to be at risk [35]. It is interesting to note that in this study, 13.3% of feed supplied to the broiler chicken were also found to be contaminated with *Salmonella* spp. Detection of *Salmonella* in poultry feed in Bangladesh is not uncommon since earlier Malorny et al. [36]; Islam et al. [32] and Al Mamun et al. [26] also reported presence of *Salmonella* in poultry feed. Our findings suggest that contaminated feed could be a potential source of *Salmonella* in poultry.

AMR is an escalating global health problem [37]. Treatment of infections caused by MDR bacteria are expensive and may be fatal. AMR can affect sustainable development goals (SDGs), especially those targeting hunger, health and economic growth [38]. MDR *Salmonella* has emerged as a major public health issue worldwide [39]. One of the classical examples of such public health issue is MDR *S. enterica* serovar Typhimurium phage type DT104, which was found to be resistant to several antibiotics, namely ampicillin, chloramphenicol, streptomycin, sulfonamides, and tetracycline [40]. The present study revealed that 97.1% to 77.1% of the isolated *Salmonella* showed resistance against commonly used antibiotics, namely tetracycline, ampicillin, streptomycin, and chloramphenicol. An alarming result was that all the tested isolates in the present study were found to be MDR. Development of antibiotic resistance in these bacteria could be a result of several factors including strong selective pressure resulting from the indiscriminate use of antibiotics [41]. Moreover, unpublished data suggested that, in many cases, these antibiotics are used as a growth promoter and are added to the poultry feed and water, respectively, by feed companies and the farmers. There is evidence of antibiotic residues in commercial feed in Bangladesh, acts as subnormal dose which also accelerates the emergence of antibiotic resistance [42]. Cloacal isolates always showed a higher resistance against different antibiotics than litter and feed isolates, which indicates that feces may play a vital role in the spread of resistant bacteria within the farm environment, including feed stored in the poultry shed.

The present study reported the detection of *tetA* (97.1%), *floR* (94.3), *bla_TEM-1_* (82.9%) and *aadA1* (77.1%) responsible for specific antibiotic resistance. The presence of several resistance genes in different samples may be due to integron that was detected in this study. Integrons are mobile genetic elements. They play important role in the transfer of clusters of genes including antibiotic resistance. Integrons could also be transferred horizontally among *Salmonella* serotypes [43,44]. *Salmonella* carrying class one integron *int1* were found positive for multiple resistance genes. In this study, 20% isolates were found positive for the presence of the *int1* gene, revealing the molecular basis of observed multidrug resistance [45]. These factors may facilitate the emergence of MDR pathogens. In many cases, resistance patterns are observed against those antimicrobials that are frequently used in veterinary practices [45,46].

MDR *Salmonella* are important to the etiology of bacterial foodborne associated deaths, particularly in the LMICs [47]. The WHO recognized *Salmonella* as one of the pathogens having severe impacts on human health [48]. Poultry and poultry products have been frequently reported to be involved in the outbreaks of salmonellosis [49]. Alarmingly, all the *Salmonella* isolated in the present study were found to be MDR including fluroquinolone-resistant *Salmonella*. The detection of MDR *Salmonella* in broiler chicken as evident in this study is of great public health concern. It is possible that these MDR *Salmonella* may transmit into the food chain in broiler meat and lead to serious illness in humans. Detection of mobile genetic elements, such as integron in these isolates make the situation more aggravated. Integrons are directly associated with resistance to antibiotics [50] and there are possibilities of their transmission to other bacterial species horizontally inside the broiler chicken gut. The present findings highlight the importance of practicing strict biosecurity, hygienic measures, proper litter management and safe storage of feed to reduce the load and spread of MDR *Salmonella* in broiler chicken and to ensure consumers’ health and safety. In addition, the implementation of one-health measure in Bangladesh is critical to early monitor and detect AMR in pathogens of zoonotic potential and to reduce the imprudent use of antibiotics in poultry farms.

## 4. Materials and Methods

### 4.1. Ethics Statement

The experimental procedures and protocols used in this study were approved by the Animal Welfare and Experimentation Ethics Committee of Bangladesh Agricultural University (approval number AWEEC/BAU/2018(20) 

### 4.2. Collection of Samples and Isolation of Salmonella

A total of 100 samples (50 cloacal swabs, 35 litter samples, 15 feed samples) from five broiler (*Gallus gallus domesticus*) farms located in Mymensingh, Bangladesh were randomly collected in July 2017 and samples were transported immediately to the Department of Microbiology and Hygiene, Bangladesh Agricultural University, while maintaining sterile and cold chain conditions. From each farm, 10 cloacal swabs, 7 litter samples and 3 feed samples were collected. Isolation and identification of *Salmonella* from the collected samples were based on culture on selective media (XLD) (Hi Media, India), Gram staining and biochemical test as was previously described [29].

### 4.3. Extraction of Bacterial Genomic DNA 

For the PCR, the genomic DNA was extracted using the boiling method [51]. In brief, initially a pure bacterial colony was mixed with 100 µL of distilled water in an Eppendorf tube followed by boiling for 10 min. After boiling the Eppendorf tube, it was immediately kept on ice to have cold shock followed by 10 min of centrifugation at 10,000 rpm. Finally, the supernatant was collected and used as a DNA template for PCR. 

### 4.4. Molecular Detection of Salmonella

*Salmonella* was detected by PCR targeting the *invA* gene as previously described [52]. Motile *Salmonella* were detected by PCR targeting the *fliC* gene specific for *S. enterica* serovar Typhimurium [53]. Primers specific for *invA* and *fliC* genes are listed in Table 5. PCR was done in the final 25 µL containing nucleus free water (5.5 µL), master mixture (Promega) (12.5 µL), forward and reverse primer (1 µL each) and DNA template (5 µL). The PCR thermal profile consisted of 95 °C and 5 min for initial denaturation, followed by 29 cycles of denaturation at 95 °C for one-minute, variable annealing temperature (Table 5) for one-minute, elongation at 72 °C for one- minute and a final extension at 72 °C for 10 min. After completion, the PCR products were analyzed by running in 1.5% agarose gel electrophoresis.

### 4.5. Antimicrobial Susceptibility Testing

The antibiogram phenotype were determined by the Kirby-Bauer disk diffusion method [54] against seven commonly used antibiotics classes, namely penicillin (ampicillin-25 µg), cephalosporin (cefixime-5 µg), amphenicol (chloramphenicol-30 µg), fluoroquinolones (ciprofloxacin-5 µg), carbapenem (ertapenem-10 µg), aminoglycoside (streptomycin-10 µg) and tetracycline (tetracycline-30 µg). The antibiogram test was performed by disk diffusion method on Mueller-Hinton agar (Hi Media, India) plates with a concentration of bacteria equivalent to 0.5 McFarland standard, incubated for 18–24 h aerobically at 37 °C. The results of the antibiogram test were recorded as sensitive, intermediately sensitive, or resistant and the diameters of the zones of inhibition were compared with the diameters of interpretative tables provided by the Clinical and Laboratory Standards Institute (CLSI) [55]. *Salmonella* isolates that were found to be resistant to multiple antimicrobials (at least 3 classes of antibiotics) were considered as MDR [56].

### 4.6. Detection of Antibiotic Resistance and Class 1 Integron Gene

All the MDR phenotypes were subjected to PCR for the detection of *aadA1*, *bla_TEM-1_*, *floR,* and *tetA* genes responsible for resistance against streptomycin, beta lactam antibiotic, amphenicol (chloramphenicol), and tetracycline respectively, and class 1 integron gene *intl1* [57,58,59,60]. The list of primers used to detect these genes are presented in Table 5.

### 4.7. Statistical Analysis

All the data was incorporated in Excel sheets (MS-2010) and analyzed by SPSS software (SPSS-24.0). Descriptive analysis was performed to calculate prevalence and the Chi-square test was performed to determine the level of significance. A *p*-value less than 0.05 (*p*-value < 0.05) was considered as statistically significant.

## Figures and Tables

**Table 1:** Antibiotic resistance patterns of *Salmonella* in broiler farm in this study.

Sample (n)	Antibiotic Resistance Pattern (%)													
	TE	*p* Value	C	*p* Value	AMP	*p* Value	S	*p* Value	CIP	*p* Value	ETP	*p* Value	CFM	*p* Value
Cloacal Swab (24)	24 (100)	<0.001	24 (100)	0.010	23 (95.8)	0.011	21 (87.5)	0.012	4 (16.7)	0.772	2 (8.3)	0.615	0 (0)	nc
Litter (9)	9 (100)		8 (88.9)		5 (55.6)		6 (66.7)		1 (11.1)		0 (0)		0 (0)	
Feed (2)	1 (50)		1 (50)		1 (50)		0 (0)		0 (0)		0 (0)		0 (0)	
Total (35)	34 (97.1)		33 (94.3)		29 (82.9)		27 (77.1)		5 (14.3)		2 (5.7)		0 (0)	

Note: TE, tetracycline; C, chloramphenicol; AMP, ampicillin; S, streptomycin; CIP, ciprofloxacin; ETP, ertapenem; CFM, cefixime; nc, not computed; n, no. of positive *Salmonella*.

**Table 2:** Multidrug resistance profile of *Salmonella* used in this study.

Isolates	No. of Antibiotic (Class)	Multidrug Profile	No. of Isolates (%)	Prevalence of MDR %
*Salmonella* (n = 35)	1 (1)	Any one of the tested antibiotics	0	100
	2 (2)	TE-AMP or other combination of two antibiotic classes	0	
	3 (3)	TE-AMP-C	28 (80)	
		TE-S-C	26 (74.28)	
	4 (4)	C-AMP-S-TE	24 (68.57)	
		CIP-AMP-TE-S	5 (14.28)	
	5 (5)	C-AMP-S-TE-CIP	1 (2.87)	
		C-AMP-S-TE-ETP	1 (2.87)	

Note: TE, tetracycline; AMP, ampicillin; C, chloramphenicol; S, streptomycin; CIP, ciprofloxacin; ETP, ertapenem; MDR, multidrug resistance.

**Table 3:** Prevalence of antibiotic resistance genes in *Salmonella* in broiler farm in the present study.

Sample (n)	Antibiotic Resistance Gene (%)									
	*terA*(TE)	*p* Value	*floR* (C)	*p* Value	*bla_TEM-1_* (AMP)	*p* Value	*aadA1* (S)	*p* Value	Class 1 Integron *intl1*	*p* Value
Cloacal Swab (24)	24 (100)	<0.001	24 (100)	0.010	23 (95.8)	0.011	21 (87.5)	0.012	6 (25)	0.517
Litter (9)	9 (100)		8 (88.9)		5 (55.6)		6 (66.7)		1 (11.1)	
Feed (2)	1 (50)		1 (50)		1 (50)		0 (0)		0 (0)	
Total (35)	34 (97.1)		33 (94.3)		29 (82.9)		27 (77.1)		7 (20)	

Note: TE, tetracycline; AMP, ampicillin; C, chloramphenicol; S, streptomycin; n, no. of positive *Salmonella*.

**Table 4:** Profiles of class 1 integron gene *intl1* carrying *Salmonella* in the present study.

Isolate number	Resistance Genes	MDR Profile
CS3	*terA, floR, bla_TEM_-_1_, aadA1*	C-AMP-S-TE
CS11	*terA, floR, bla_TEM_-_1_, aadA1*	C-AMP-S-TE-ETP
CS19	*terA, floR, bla_TEM_-_1_*	C-AMP-S-TE
CS22	*terA, floR, bla_TEM_-_1_, aadA1*	C-AMP-S-TE
CS41	*terA, floR, bla_TEM_-_1_, aadA1*	C-AMP-S-TE-CIP
CS43	*terA, floR, bla_TEM_-_1_, aadA1*	C-AMP-S-TE
L8	*terA, floR, aadA1*	C-AMP-S-TE

Note: CS, cloacal swab; L, litter, MDR, multidrug resistance; C, chloramphenicol; TE, tetracycline; AMP, ampicillin; S, streptomycin; CIP, ciprofloxacin; ETP, ertapenem.

**Table 5:** PCR primers with sequence used in this study.

Gene	Primer Sequence	Amplicon Size (bp)	Annealing Temperature	Reference
*invA-F*	CGGTGGTTTTAAGCGTACTCT T	796	58	[52]
*invA-R*	CGAATATGCTCCACAAGGTTA			
*Flic-C-F*	CCCGCTTACAGGTGGACTAC	433	58	[53]
*Flic-C-R*	AGCGGGTTT TCGGTGGTTGT			
*aadA1-F*	TATCAGAGGTAGTTG GCGTCAT	484	55	[57]
*aadA1-R*	GTTCCATAGCGTTAAGGTTTCATT			
*bla_TEM-1_-F*	CATTTCCGTGTCGCCCTTAT	793	56	[58]
*bla_TEM-1_-R*	TCCATAGTTGCCTGACTCCC			
*floR-F*	AACCCGCCCTCTGGATCAAGTCAA	548	62	[57]
*floR-R*	CAAATCACGGGCCACGCTGTATC			
*tetA-F*	GGTTCACTCGAACGACGTCA	577	57	[59]
*tetA-R*	CTGTCCGACAAGTTGCATGA			
*intl1 A-F*	GGCATCCAAGCAGCAAGC	2000	55	[60]
*intl1 A-R:*	AAG CAG ACT TGA CCT GAT			
